# A Test of the Long‐Term Efficiency of Genetic Rescue With Drosophila melanogaster

**DOI:** 10.1111/mec.17690

**Published:** 2025-02-18

**Authors:** Noelia Pérez‐Pereira, Daniel Kleinman‐Ruiz, Aurora García‐Dorado, Humberto Quesada, Armando Caballero

**Affiliations:** ^1^ Centro de Investigación Mariña Universidade de Vigo, Facultade de Bioloxía Vigo Spain; ^2^ Universidad de Sevilla Sevilla Spain; ^3^ Departamento de Genética, Facultad de Ciencias Biológicas Universidad Complutense Madrid Spain

**Keywords:** deleterious mutations, fitness, genetic diversity, genetic purging, hybrid vigour, inbreeding depression, inbreeding load

## Abstract

Genetic rescue is considered a promising but underutilised conservation strategy to mitigate inbreeding depression and restore genetic diversity. Yet, empirical evidence supporting its long‐term efficacy is limited to studies investigating short‐term effects. Here, we conducted an experiment with *Drosophila* to test the long‐term efficiency of genetic rescue across generations. A wild population was captured to found a genetically diverse mass‐bred base population (BP) in the laboratory. Smaller populations of 50 individuals each (N50) were then founded from the BP and maintained for 31 generations. Three sets of lines of eight individuals each were founded from these N50 populations: non‐rescued (control), rescued with BP males, and rescued with N50 males. These lines were maintained for 33 generations. Pupae productivity analysis showed substantial purging in N50 populations and adaptation to laboratory conditions in the BP. Rescued‐BP lines showed a higher productivity and lower extinction rates compared to non‐rescued lines. Whole‐genome sequencing of individuals from a non‐rescued line and a rescued‐BP line revealed fewer deleterious alleles, lower genetic diversity, and higher inbreeding in the rescued line, suggesting efficient rescue. Our results enlighten the importance of introducing new genetic variation allowing for adaptation to increase survival even in small populations despite the simultaneous introduction of an inbreeding load, particularly when facing global changes affecting environmental conditions of both donor and recipient populations.

## 1 Introduction

Genetic rescue is aimed at reducing the extinction probability of endangered populations by introducing migrant individuals (Bell et al. 2019). This strategy has proven successful in multiple cases (Vilà et al. 2002; Fredrickson et al. 2007; Johnson et al. 2010; Åkesson et al. 2016; Weeks et al. 2017; Hasselgren et al. 2018; Ralls et al. 2020) and is considered a promising approach in Conservation Biology (Waller 2015; Tallmon 2017). However, most studies on genetic rescue follow effects for only a few generations post‐rescue (Whiteley et al. 2015; Fitzpatrick et al. 2020). Concerns have been raised based on the case of the Isle Royale wolves ( *Canis lupus* ), where the genetic input of a single migrant wolf from a large mainland population rapidly spread among the resident population (Hoy et al. 2023). This rapid integration, attributed to the high breeding vigour of the offspring, potentially led to increased inbreeding and an associated fitness decline, ultimately resulting in population extirpation (Hedrick et al. 2014, 2017, 2019; but see Ralls et al. 2020). Thus, despite multiple studies endorsing genetic rescue (Frankham 2015; Kolodny et al. 2019), its medium‐ to long‐term consequences remain uncertain (Hedrick and Fredrickson 2010; Hedrick and García‐Dorado 2016; Bell et al. 2019; Ralls et al. 2020; Pérez‐Pereira et al. 2022; Jørgensen et al. 2022).

From a genetic standpoint, the primary factor driving extinction in small endangered populations is inbreeding depression causing reduced fitness (O'Grady et al. 2006; Allendorf et al. 2013; Frankham et al. 2014). This occurs due to the expression of the inbreeding load, the burden of recessive deleterious effects hidden in heterozygous condition (Morton et al. 1956), and manifested by inbreeding. In stable populations, the inbreeding load is expected to be larger for populations of large effective size (García‐Dorado 2003, 2007; Hedrick and García‐Dorado 2016). Thus, a historically large population can appear genetically healthy by exhibiting high average fitness, but individuals from such populations are expected to be heterozygous for many rare, partially recessive deleterious alleles. In small, endangered populations, the reduction of effective population size leads to an increase in both genetic drift and inbreeding over successive generations. However, this also intensifies natural selection against (partially) recessive deleterious alleles, a process known as genetic purging (García‐Dorado 2012). The efficiency of purging in removing inbreeding load remains a highly debated issue. Fast inbreeding, as observed in scenarios of sustained full‐sib mating or in populations with fewer than 10 individuals, is widely recognised to promote purging of lethal or severely deleterious mutations, but it is less effective against mildly deleterious ones (Hedrick 1994; Wang et al. 1999; Pekkala et al. 2012; Bersabé and García‐Dorado 2013; Caballero et al. 2017). With respect to slow inbreeding (populations with effective sizes of 10s of individuals, or large populations with a low proportion of non‐random mating), the experimental evidence on purging is more controversial. Some studies support its effectiveness (e.g., Kleinman‐Ruiz et al. 2022; Khan et al. 2021; Roessler et al. 2019), while others question it (e.g., Taylor et al. 2024; Zeitler et al. 2023; Grossen et al. 2020; Liu et al. 2017). However, the magnitude of purging depends on specific conditions (Bijlsma et al. 1999; García‐Dorado 2012), and long‐term experiments carefully designed for that purpose have shown their efficiency in removing mildly deleterious mutations (López‐Cortegano et al. 2016; Pérez‐Pereira et al. 2021).

Introducing migrant individuals from large, genetically healthy populations into small endangered ones often results in population recovery over a few generations (Whiteley et al. 2015; Ralls et al. 2020). However, as indicated above, migrants from historically large donor populations can carry many partially recessive deleterious alleles that were rare in the source population. While these alleles may not cause an initial fitness decline in the recipient population, they can contribute to its inbreeding load, which could elevate the risk of future inbreeding depression (Hedrick and García‐Dorado 2016). Thus, while using large donor populations can provide short‐term benefits, it could theoretically heighten the risk of extinction in very small endangered populations due to potential future inbreeding depression. Therefore, the success of genetic rescue programs in reducing the extinction risk associated with inbreeding depression depends on balancing two opposing effects of gene flow. On one hand, migrants reduce inbreeding, leading to an increase in fitness due to hybrid vigour or heterosis. This occurs because the introduction of beneficial alleles from migrants can replace deleterious alleles that have reached high frequency in the endangered population. On the other hand, migrants carry their own inbreeding load, which can be expected to be particularly large if migrants came from large unpurged populations. This hidden inbreeding load can contribute to future inbreeding depression in the endangered population, although it can be mitigated by subsequent purging. Thus, the success of genetic rescue programs critically depends on the purging that occurs in both the donor and recipient populations. Other factors may also impact a rescue program, such as the potential benefits of introducing new adaptive mutations from the donor population and the risk of outbreeding depression, if the populations are adapted to different environments and/or have been separated for many generations (Frankham et al. 2011; Ralls et al. 2020).

A recent simulation study (Pérez‐Pereira et al. 2022) indicated that, for models considering inbreeding load and genetic purging due to partially recessive deleterious mutations, genetic rescue may have undesired long‐term consequences under certain particular scenarios. These occur when migrants from non‐purged large donor populations introduce substantial inbreeding load into very small populations that have already purged most of their own inbreeding load. In order to test this possibility and to investigate the long‐term efficiency of genetic rescue, we carried out a replicated experiment with the model species *Drosophila melanogaster* in which lines of eight individuals arising from moderate‐sized populations (where substantial purging is expected to have taken place) were rescued with individuals from the large laboratory population of origin or from derived populations of smaller size. Our results show that genetic rescue is efficient in reducing the probability of extinction of the small rescued populations, and that the genome of one analysed rescued line carried fewer deleterious alleles than that of a non‐rescued line. However, the rescued line showed less genetic diversity and more inbreeding than the non‐rescued one, suggesting that selection could have taken place more intensively in the rescued line.

## 2 Material and Methods

### 2.1 Experimental Design

**FIGURE 1:**
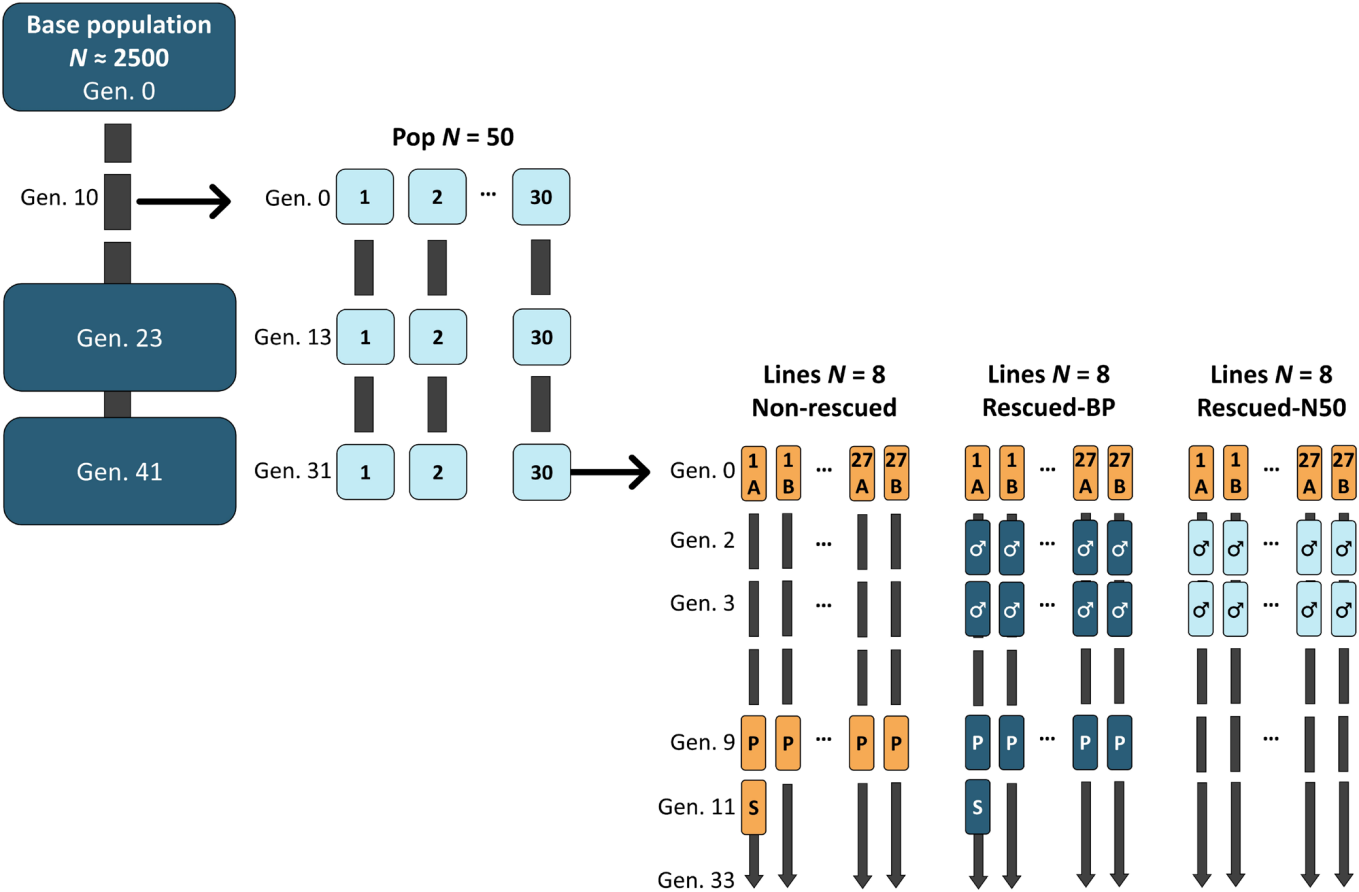
Design of the experiment. A large base population (BP) was founded and maintained in the laboratory with about 2500 individuals. Thirty populations of census size *N* = 50 (N50) individuals were founded from the base population at the 10th generation and maintained for 31 generations. At generations 23 (BP) and 13 (N50), and at generations 41 (BP) and 31 (N50), pupae productivity was evaluated simultaneously in these populations. At generation 31 of the N50 populations, small lines of *N* = 8 individuals were founded from each remaining one (three sets of two lines, A and B, per N50 population). A set of the lines was maintained as a non‐rescued control, whereas the other sets were rescued by the introgression of a single male in two consecutive generations from an N50 population different from the one of origin (rescued‐N50), or from the original large base population (rescued‐BP). At generation 7 of the lines, the remaining lines were duplicated. At generation 9, the non‐rescued and the rescued‐BP sets were also evaluated for pupae productivity (P). And at generation 11, a single non‐rescued line and a single rescued‐BP line were analysed by whole genome sequencing (S) of 12 males from each of them. All lines were maintained for a total of 33 generations to count the number of surviving lines in the three sets.

About 1000 gravid females of *Drosophila melanogaster* were captured from a wild population of the locality of Moreira (Pontevedra, Galicia, Spain) in October 2018 and placed in vials with a single female per vial. The progeny of all females was anesthetised and mixed in an empty bottle, and 32 samples of 40 males and 40 females were taken and introduced in 32 bottles which were numbered from 1 to 32. The population founded (base population, or BP, hereafter) was maintained thereafter by circular mixing of the bottles so that, for each generation (every 2 weeks), each bottle *i* was founded with approximately 40 flies from the offspring of bottle *i* plus 40 flies from the offspring of bottle *i* + 1 (*i* = 1 in the case of bottle 32). Before new offspring emerged, parents were removed to avoid overlapping generations. Thus, the BP population was maintained with about 2500 individuals (Figure 1). Throughout the experiments, flies were maintained in a room chamber with constant temperature (25°C) and permanent lighting, except when handling flies. At generation 10, a series of 30 populations of smaller size (*N* = 50, N50 hereafter; 25 individuals of each sex) were established. Each N50 population was founded by randomly sampling 1–2 individuals from each of the BP bottles and maintained in a single bottle with this same size (25 males and 25 mated females) for 31 generations. A Drosophila population with a size similar to that of the N50 populations had been shown to be efficiently purged from deleterious variation after a similar period of time by López‐Cortegano et al. (2016), in agreement with theoretical predictions and simulation results (see Pérez‐Pereira et al. 2022). At generation 31, three N50 populations had gone extinct, and from each of the remaining 27 populations, six lines (vials) were founded with *N* = 8 individuals (N8) each (four males and four females) to obtain three sets of lines. A set formed by two vials from each N50 population (vials A and B, i.e., a total of 54 lines maintained independently) was considered as a non‐rescued control. The second and third sets of 54 lines were rescued by introducing a single male in each vial at generation 2 and another male at generation 3. One set was rescued with males from a N50 population different from the population of origin (rescued‐N50 lines), and the other set was rescued with males from the base population (rescued‐BP lines). In the two generations of introgression, all male and female flies were assured to be virgin before mating, so that the introduced male would compete in equal conditions with the other four males of the vial to mate the virgin females. For the remaining generations, the four males and females were not necessarily virgin. Because the loss of lines was initially quick, all remnant lines from the three sets were duplicated at generation 7. The three sets of lines were maintained for 33 generations, and the number of line extinctions (when no progeny, or only progeny of a single sex, was obtained) was counted across time.

### 2.2 Evaluation of Fitness and Inbreeding Load

**FIGURE 2:**
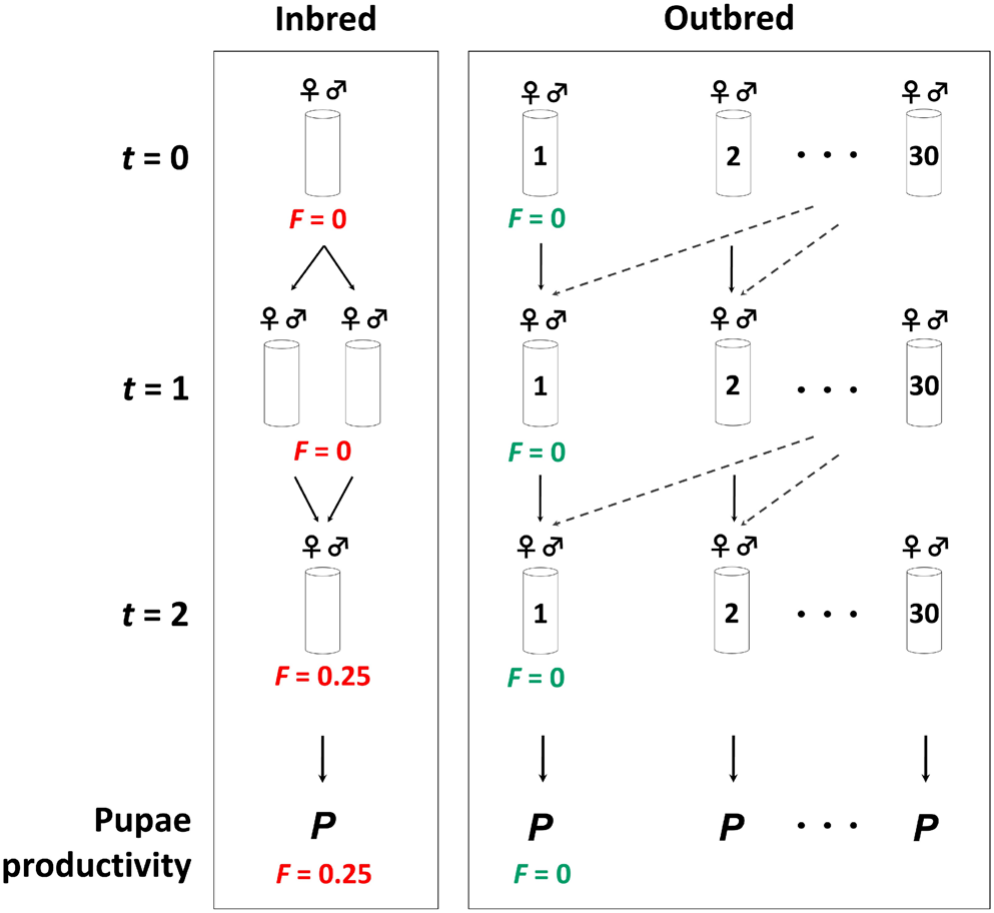
Design of experiments to estimate pupae productivity (*P*) and inbreeding depression in the base population (BP) and the *N* = 50 populations (N50). At generation 41 (BP) and 31 (N50), mating pairs were evaluated for pupae productivity following two schemes, one avoiding inbred mating (*F* = 0) in two consecutive generations, and another carrying out full‐sib mating in generation *t* = 1 and cousin mating in *t* = 2, so as to obtain an expected inbreeding coefficient (*F*) of 0.25 at both generations. From the average pupae productivity evaluated in the last generation, the inbreeding depression rate was estimated. At generations 23 (BP) and 13 (N50), the analysis was the same, but only for the outbred scheme.

We evaluated fitness simultaneously in the base population BP (generations 23 and 41) and the N50 populations (generations 13 and 31). The fitness measure was pupae productivity (*P*) estimated as the average number of pupae produced per mating pair 11 days after mating. This trait includes mating success, fecundity and survival to the pupae stage, thus being a proxy for fitness in moderately competitive conditions. A total of eight virgin females and eight males were sampled from each of the bottles of the BP and the N50 populations to generate pair mating in individual vials (generation *t* = 0 in Figure 2). For the evaluation of generations 41 (BP) and 31 (N50), which is the time of founding of the N8 lines, the offspring was used to generate two schemes: an inbred one, in which full‐sib couples were mated in individual vials, and an outbred one, in which a female from vial *i* was mated with a male from a randomly chosen vial different from vial *i*. At the next generation (*t* = 1), in the outbred scheme, mating between unrelated parents was carried out so that the expected inbreeding coefficient was zero. In the inbred scheme, mating between cousins was carried out so that the final expected inbreeding coefficient in the produced eggs (generation *t* = 3) was the same as that for the laying females (generation 2), that is, *F* = 0.25. The results were based on the pupae productivity of 81 and 56 vials for outbred and inbred schemes, respectively, for the BP population, and 48 and 40 vials, respectively, for the N50 populations. For the evaluation of generations 23 (BP) and 13 (N50) only the outbred scheme was carried out, with pupae productivity obtained from 80 vials for the BP population and 134 vials for the N50 populations.

The inbreeding load was estimated as the inbreeding depression rate (*δ*, the percent reduction of the mean expected for each 0.01 increase in inbreeding coefficient) at generations 41 (BP) and 31 (N50). It was calculated as $\delta =\frac{\left[\ln\left(\frac{P_{O}}{P_{I}}\right)\right]}{F}$, where *P*_*O*_ and *P*_*I*_ are the average productivities of outbred and inbred schemes, respectively, and *F* is the expected inbreeding coefficient of parental and progeny generations (*F* = 0.25). Bootstrap errors were obtained for each estimate of *δ* using the function ‘sample’ in R. For each estimate, 1000 samples of the same size as the original productivity data (inbred and outbred) were taken with replacement. For each sample, *δ* was obtained as above and the standard deviation of the 1000 measures was calculated.

We also evaluated pupae productivity at generation 9 of the N8 non‐rescued lines and of those rescued by individuals from the base population (rescued‐BP) following the scheme of Figure S1. In this case, between one and eight virgin females and males were sampled from each of the lines available, to generate pair mating in individual vials (generation *t* = 0 in Figure S1). We performed an inbred scheme with the aim of ascertaining whether inbreeding was more detrimental in rescued lines due to the inbreeding load introduced during rescue. In this scheme, full‐sib couples were mated in individual vials for two consecutive generations. We also performed an outbred scheme where females from each line were mated with males from a randomly chosen line different from the females' line. The aim of this scheme was to evaluate rescuing effects on fitness that were not ascribed to hybrid vigour. Pupae productivity was finally measured in 49 and 50 vials for outbred (line crossing) and inbred non‐rescued lines, respectively, and in 61 and 56 vials for outbred and inbred rescued‐BP lines, respectively. This procedure does not allow estimating the inbreeding load in N8 lines because outbred mating were made with individuals from different lines, but it can be used to evaluate the effects of rescue on the whole set of lines. Due to the marked asymmetry in the distribution of the mean productivity of the lines, overall means were contrasted using the two‐sided Mann–Whitney non‐parametric test.

### 2.3 DNA Extraction, Sequencing and SNP Calling

Twelve males from a randomly chosen non‐rescued line and 12 from a rescued‐BP line were sampled at generation 11, frozen with liquid nitrogen, and individually stored at −80°C until DNA extraction. DNA was extracted using the Gentra Puregene Cell Kit (Qiagen) with some modifications, including an RNase treatment. The Nextera XT DNA library preparation and subsequent genome sequencing were carried out at the NimGenetics Genomics Service (Madrid, Spain), where a single Illumina library was prepared for each individual fly. The genomes of these 24 individuals were sequenced on an Illumina Novaseq 6000 instrument (2 × 150 bp paired‐end) to an average read depth of approximately 50×.

Paired‐end reads were processed from the FASTQ files to generate Variant Call Format (VCF) files containing data for filtered biallelic autosomic SNPs. Initially, the adequate quality of raw reads was confirmed using FastQC (Andrews 2010); adapters were then removed using Trimmomatic (Bolger et al. 2014) with the Nextera adapter list. Quality and size filtering of the reads were performed with ERNE‐FILTER (Del Fabbro et al. 2013) using default settings except *–min‐size* = 36. The filtered reads were then mapped to the *D. melanogaster* reference genome version 6.14 from Flybase (www.flybase.org/) using BWA‐MEM (Li 2013). PCR duplicates were removed, and alignments were filtered with a minimum mapping quality score of 30 using SAMtools (Danecek et al. 2021). Next, we used picard‐tools (Broad Institute 2019) to add read groups, and the Genome Analysis Toolkit (GATK; van der Auwera and O'Connor 2020) v3.8 to perform a multirealignment with the purpose of fixing potential alignment artefacts. The overall quality of the aligned and processed reads was assessed using Qualimap (Okonechnikov et al. 2016). Variant calling was performed with the HaplotypeCaller tool from GATK (van der Auwera and O'Connor 2020). The resulting GVCF files were merged with the GATK CombineGVCFs tool, and genotype information was extracted using the GATK GenotypeGVCFs tool. For the derived count analysis, we kept coding variants found in the orthologous regions between *D. melanogaster* , *D. simulans* , and *D. yakuba*, as defined in the release FB2021_06 version of a precomputed file with records of all known *Drosophila* orthologs taken from https://flybase.org. To refine the dataset, multiallelic and monomorphic SNPs, indels, and SNPs within 10 bp of an indel were excluded using BCFtools (Danecek et al. 2021). We also discarded those SNPs with missingness > 15% (i.e., those not covered in more than three individuals), plus those with very high or low overall coverage (beyond the average ± 2.5 SD). Additionally, highly repetitive regions and low‐information sites were filtered out using bedtools (Quinlan and Hall 2010). The remaining SNPs were hard‐filtered using the GATK VariantFiltration tool, applying the recommended presets. PED and MAP files were generated from the filtered VCF file using PLINK (version 1.9; Purcell et al. 2007). The final dataset included 207,426 SNPs from autosomal chromosomes ready for analysis. For the Runs‐of‐Homozygosity (ROH) analysis (see next section) we adopted an alternative, less restrictive filtering that did not discard non‐coding regions. After excluding multiallelic and monomorphic SNPs, indels, and variants lying in the small chromosome 4 using BCFtools, a total of 392,395 and 349,716 SNPs remained in the non‐rescued and rescued‐BP datasets, respectively.

### 2.4 Genomic Diversity and Runs‐Of‐Homozygosity

We estimated different diversity statistics with the software DnaSP6 (Rozas et al. 2017), namely: the nucleotide diversity (*π*); Watterson's *θ*, which gives the number of segregating sites corrected by the number of sequences; Eta, the total number of mutations; the number of heterozygous positions per individual; and Tajima's *D*, which measures the deviation from neutrality in the frequency spectrum. Additionally, Runs‐of‐Homozygosity (ROH) segments were determined using the software PLINK. The minimum number of SNPs to define a ROH was obtained with the method of Lencz et al. (2007) and Purfield et al. (2017), assuming a percentage of false positives of 0.05, being 32 for the non‐rescued line and 24 for the rescued‐BP line, and the minimum length considered for a ROH was 100 kb. Minor allele frequency and linkage disequilibrium pruning were not carried out in order to avoid missing ROH, as recommended by Meyermans et al. (2020). The other parameters used were: a minimum density of 1 SNP per 50 kb, a homozygous gap of 50 kb, a scanning window of 30 SNPs, and a threshold window of 0.05, with the remaining parameters as default by PLINK. Alternative analyses were also made changing the above parameters in turn: a minimum density of 1 SNP per 200 kb, a homozygous gap of 200 kb, a scanning window of 60 SNPs, or a threshold window of 0.01. The average inbreeding coefficient of individuals was obtained as $F_{\mathrm{ROH}}=\frac{{\Sigma L}_{\mathrm{ROH}}}{L}$ (Broman and Weber 1999; McQuillan et al. 2008), where Σ*L*_*ROH*_ is the sum of the lengths of all ROH found in an individual, and *L* is the autosomic genome length analysed (110 Mb).

### 2.5 Inference of the Ancestral and Derived States

To carry out variant polarisation, we designed a detailed procedural framework. The central task of distinguishing between ancestral and derived states was performed through the use of est‐sfs v2.03 (Keightley and Jackson 2018). This software uses a maximum likelihood approach to estimate the unfolded site frequency spectrum specific to a focal species, integrating data from multiple outgroup species. It delivers nearly unbiased probabilities of ancestral and derived states at each genomic site. For this analysis, we utilised as outgroup sequence data from two *Drosophila* species obtained from the NCBI Sequence Read Archive (SRA): *D. simulans* (accession code SRR6425999) and *D. yakuba* (accession code SRR6426004).

We obtained consensus states for *D. simulans* and *D. yakuba* by extracting haploid sequences (in FASTA format) aligned to the *D. melanogaster* reference genome. This involved using samtools mpileup (Li et al. 2009), with parameters ‐s ‐q30 to filter out sites with mapping quality below 30, and pu2fa from Chrom‐Compare (https://github.com/Paleogenomics/Chrom‐Compare) with ‐b 1 to manage haploidisation at polymorphic sites. Next, a custom multi‐step bash script was developed to construct the input file for est‐sfs. We executed est‐sfs v2.03 chromosome by chromosome using the rate‐6 model, and parsed the program output using another custom bash script, accepting inferences if the probability of the major allele being ancestral was ≥ 0.9 (defining it as ancestral), or < 0.1 (defining it as derived). The *D. melanogaster* reference genome FASTA file was then transformed into an ancestral state reference genome using a custom bash script. Finally, fill‐aa from VCFtools v0.1.17 (Danecek et al. 2011) was utilised to incorporate the inferred ancestral state into the INFO/AA (ancestral allele) field of the VCF file, followed by VcfFilterJdk (Lindenbaum and Redon 2018) to reformat the file from a reference/alternate (unpolarised) state to an ancestral/derived (polarised) allele definition.

### 2.6 SNP Annotation and Deleterious Mutation Prediction

We used ANNOVAR v4.19 (Wang et al. 2010) for the annotation of genetic variants, enabling their classification into intergenic, intronic, and coding sequences (CDS). Within the CDS category, mutations were further divided into synonymous, missense (non‐synonymous), and loss‐of‐function (LoF) mutations, the latter including stop‐gain and stop‐loss changes. We created a custom annotation database informed by our inferred ancestral state, utilising the ancestral genome FASTA and the refGene gene model from the Gene Transfer Format (GTF) file of the *D. melanogaster* reference genome release 6, obtained from UCSC. For each gene, we retained only the primary isoform, identified as the longest transcript.

Missense mutations exhibit a broad spectrum of effects on fitness (Eyre‐Walker et al. 2006). To pinpoint the most potentially harmful mutations, we employed SIFT4G v2.4 (Vaser et al. 2016) and Provean v1.1.5 (Choi and Chan 2015). These tools assess the functional impact of amino acid changes based on sequence similarity and the physicochemical properties of amino acids, with Provean also taking sequence context into account. We ran SIFT following the prescribed guidelines, using a custom database based on our ancestral genome with the UniProt ref90 protein database (Suzek et al. 2015), applying the default threshold (SIFT score ≤ 0.05) to identify variants as potentially deleterious. For the Provean analysis, we followed the methodology detailed by Kleinman‐Ruiz et al. (2022). Specifically, we employed bedtools getfasta (Quinlan and Hall 2010) to extract all coding sequences from the ancestral genome FASTA file for genes containing missense variants in our dataset. For genes located on the reverse strand, we generated the reverse complementary sequence using a custom Linux script. These nucleotide sequences were then translated into amino acid sequences using the Translate web tool from the ExPASy Bioinformatics Resources Portal (Artimo et al. 2012). For each missense variant, we extracted the ancestral and derived amino acids from the annotated VCF file and calculated the Provean deleteriousness using default parameters, classifying variants with a score of −2.5 or lower as deleterious. To ensure high confidence in identifying high‐impact mutations, we retained only those variants that were simultaneously classified as deleterious by both SIFT and Provean.

### 2.7 Obtaining Derived Counts of Variants

The individual derived count at any given site, including those segregating and those alternatively fixed between the two lines, refers to the total number of copies that support the derived allele, as inferred by GATK (i.e., 1 in heterozygotes, and 2 in homozygotes). For every individual, we recorded the derived count across different annotation categories and normalised these counts by the derived count in the putatively neutral four‐fold synonymous category to mitigate technical noise. To facilitate consistent comparisons across categories, we further standardised these normalised ratios by dividing them by those obtained for the non‐rescued lines, thus enabling a uniform scale for visualisation.

### 2.8 Relating Recombination Rate to Deleterious Predictions

Deleterious mutations are expected to be more effectively purged in regions of high recombination compared to those with low recombination rates (e.g., Bersabé et al. 2016). To test this hypothesis, we categorised variants into quartiles based on recombination rates. We utilised the *D. melanogaster* recombination rates in cM/Mb from Comeron et al. (2012) at 100 kb intervals along each chromosome arm. We used the FlyBase Coordinates Converter tool (Gramates et al. 2022) to convert coordinates to the appropriate reference genome version. Bedtools intersect (Quinlan and Hall 2010) was used to obtain the recombination rate of each SNP in our dataset, which were then ranked. Variants in the highest quartile were classified as “high recombination” while those in the lowest quartile were labelled as “low recombination”. We then recorded the derived count of each of these two classes as previously outlined.

## 3 Results

### 3.1 Productivity and Inbreeding Load

Table 1 shows the average pupae productivity and the estimates of inbreeding load in the base population (BP) and the N50 populations. The results show that a substantial purging of the inbreeding load (*δ*) occurred in the N50 populations during the first 31 generations after their foundation, with an estimated *δ* (0.83) less than half the synchronous estimate for the base population (1.99). The mean productivity of the N50 populations at generation 13 (67.18 pupae) was very close to that of the BP population (69.18 pupae). However, the productivity of the N50 populations by generation 31 (65.58 pupae at the time of the foundation of the lines) was nearly 30% lower than that of the BP (90.60 pupae), which was likely due to a large increase in the productivity of the base population.

**TABLE 1:** Average pupae productivity (*P*_*O*_ and *P*_*I*_) and estimated inbreeding load (*δ*) for the replicated vials of the base population (BP) and the 27 populations with *N* = 50 (N50) individuals derived from it, measured simultaneously at generation 23 (BP) and 13 (N50), and at generation 41 (BP) and 31 (N50). The latter is the generation at which the small lines of *N* = 8 individuals were founded from the N50 populations. *P*_*O*_ and *P*_*I*_ are the average productivities of outbred (*F* = 0) and inbred (*F* = 0. 25) individuals, respectively, based on *n* mating pairs, and obtained by the procedure described in Figure 2.

	*P*_*O*_	*P*_*I*_	*δ*
**Base population (BP)**			
Gen. 23	69.18 ± 3.87 (*n* = 80)		
Gen. 41	90.60 ± 4.25 (*n* = 81)	55.09 ± 4.65 (*n* = 56)	1.990 ± 0.397
**N50 populations**			
Gen 13 (gen. 23 of BP)	67.18 ± 2.96 (*n* = 134)		
Gen 31 (gen. 41 of BP)	65.58 ± 4.81 (*n* = 48)	53.35 ± 5.81 (*n* = 40)	0.826 ± 0.540

**FIGURE 3:**
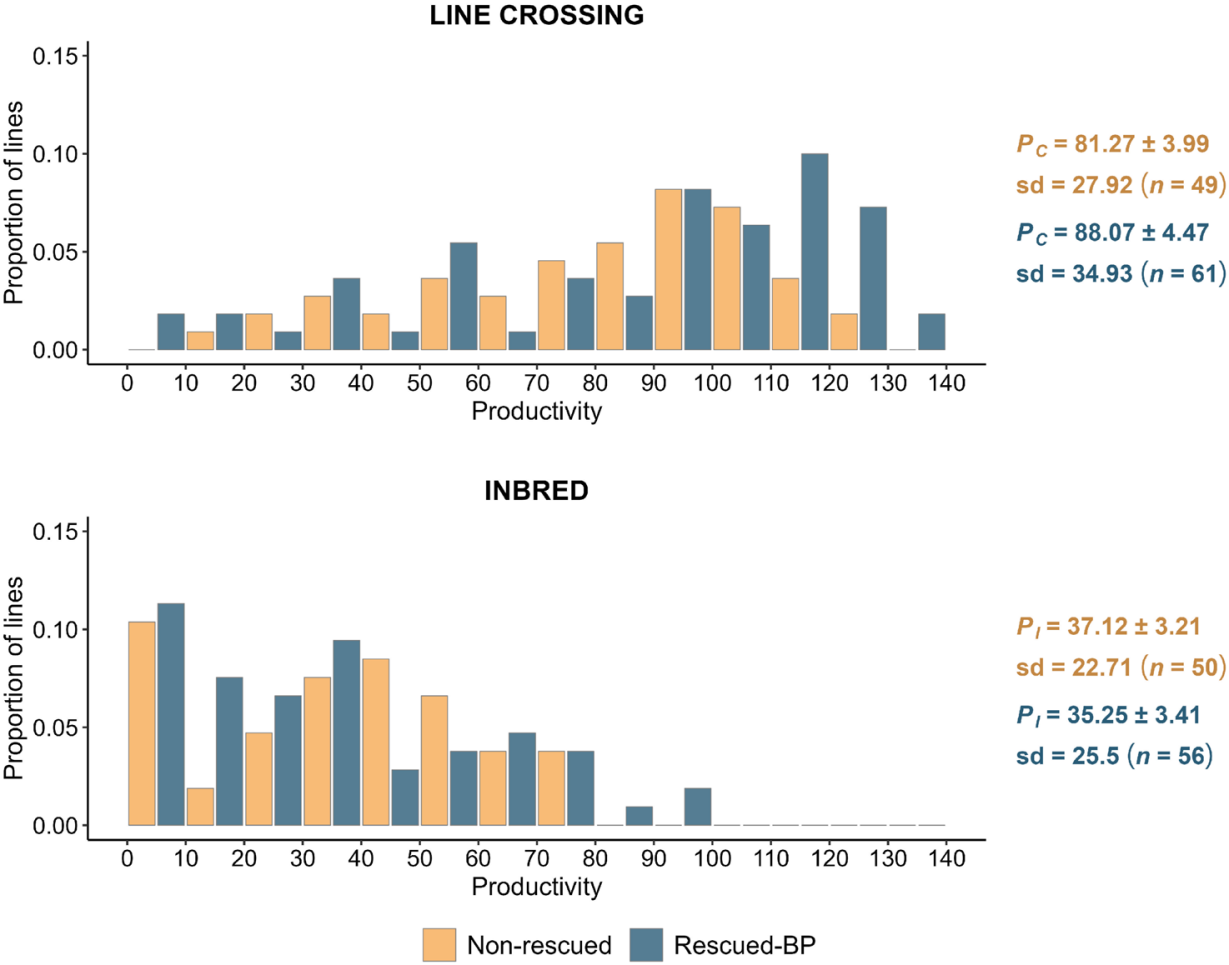
Distribution of average pupae productivity for the crosses between N8 lines (*P*_*C*_) at generation 9 and in the inbred crosses (*P*_*I*_) from non‐rescued (orange) lines and lines rescued by the base population (blue; Rescued‐BP). Mean productivities (*P*_*C*_, *P*_*I*_) and standard deviation (sd) of productivities are shown in the figure along with the number of pairs evaluated (*n*) and the associated standard errors.

Figure 3 shows the distribution of the average productivities of the non‐rescued and rescued‐BP lines at generation 9. The mean productivity of the crosses between lines (*P*_*C*_) was larger for the rescued‐BP lines than for the non‐rescued lines (Mann–Whitney test *p* < 0.037). The standard deviations of the productivities were also larger in the rescued lines than in the non‐rescued ones. Productivities for inbred individuals were similar for rescued‐BP and non‐rescued lines, the latter being somewhat larger than the former, but not significantly so (Mann–Whitney test *p* < 0.324).

The sequenced rescued‐BP line showed a clearly lower genetic diversity than the sequenced non‐rescued one (Table 2). The nucleotide diversity (*π*), Watterson's *θ*, Eta statistic, and the number of heterozygous positions per individual were all lower in the rescued line than in the non‐rescued one. In addition, Tajima's *D* was higher in the rescued line than in the non‐rescued one, indicating an excess of mutations at intermediate‐high frequencies in the former, which could be due to the introgression of male genomes from the BP population.

**TABLE 2:** Statistics obtained from the whole genome sequencing of 12 males of a non‐rescued line and 12 males from a rescued‐BP line. *π*: Nucleotide diversity per site; Heterozygous positions: Obtained by counting the number of heterozygous sites in a single individual; *θ* Watterson: Number of segregating sites corrected by the number of sequences; Eta: Total number of mutations; Tajima's *D*: A measure of the deviation from neutrality in the frequency spectrum. A positive value indicates an excess of mutations at intermediate–high frequencies; *F*_*ROH*_: Average coefficient of inbreeding of the individuals obtained from the proportion of the genome in Runs of Homozygosity; Average number and average length of ROH per individual.

	Non‐rescued	Rescued‐BP
*π*	0.000915	0.000714
Heterozygous positions	251,079	165,063
*θ* Watterson	16742.27	11003.96
Eta	62520.50	41092.00
Tajima's D	1.7239	2.672
*F*_*ROH*_	0.406 ± 0.020	0.450 ± 0.021
Number of ROH per individual	105.67 ± 3.71	121.08 ± 5.30
Length of ROH (kb)	421.62 ± 11.22	408.52 ± 3.58

**FIGURE 4:**
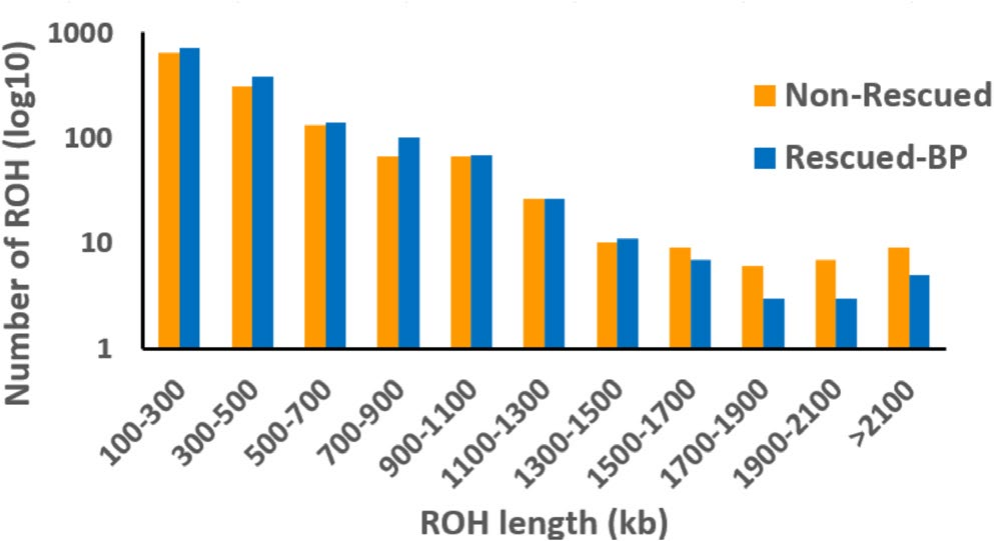
Distribution of Runs of Homozygosity (ROH) found in the non‐rescued and rescued‐BP lines as a function of their length in kilobases.

Additionally, the rescued‐BP line exhibited a significantly higher number of ROH per individual (121.08 ± 5.30) than the non‐rescued line (105.67 ± 3.71), despite having similar average ROH lengths (408.52 ± 3.58 and 421.62 ± 11.22, respectively). The average inbreeding coefficient from ROH (*F*_*ROH*_) was significantly higher in the rescued line (0.450 ± 0.021) than in the non‐rescued one (0.406 ± 0.020), in agreement with the above results indicating lower genetic diversity in the former (individual *F*_*ROH*_ values are plotted in Figure S2). Additional ROH analyses considering different PLINK options (Table S1) led to results very similar to those presented above. The distribution of ROH in the two lines is shown in Figure 4. The rescued line had a larger number of short ROH (< 1.5 Mb), whereas longer ROH (> 1.5 Mb) were more numerous in the non‐rescued line.

### 3.2 Line Extinctions

**FIGURE 5:**
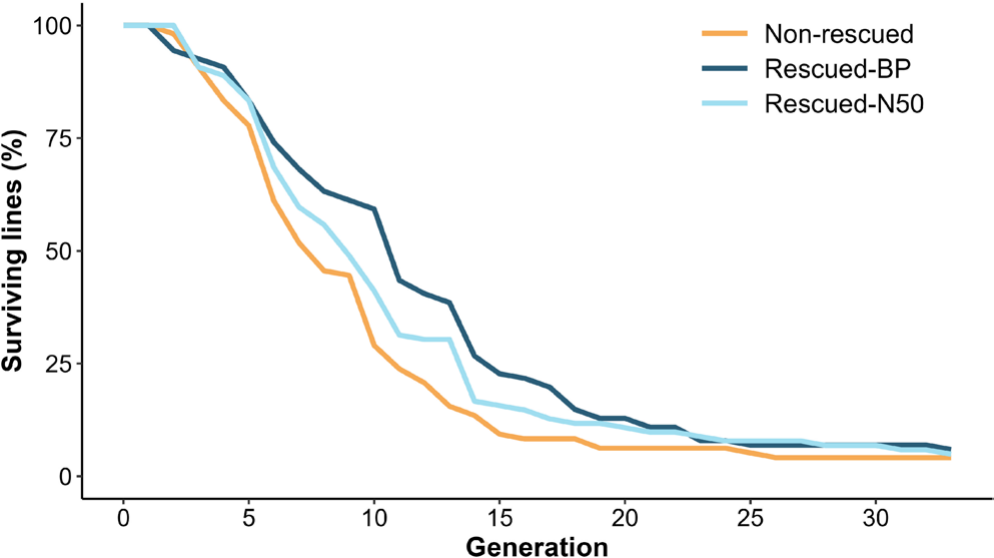
Percentage of surviving lines of size *N* = 8 individuals across generations. Non‐rescued lines, lines rescued by the *N* = 50 populations (Rescued‐N50) and lines rescued by the base population (Rescued‐BP) are shown.

Figure 5 shows the proportion of surviving lines across generations. Throughout the experiment, the rescued‐BP lines maintained a higher proportion of surviving lines compared to both the rescued‐N50 lines and the non‐rescued lines. This was ascribed to a smaller rate of extinction in the rescued‐BP lines during the first third of the experiment (0.07% for rescued‐BP, compared to 0.10% and 0.12% for rescued‐N50 and non‐rescued, respectively; Table S2). The smaller extinction risk during the second third of the experiment corresponded to the rescued‐N50 lines, while the non‐rescued ones showed the smallest extinction rate during the last third of the experiment (Table S2). However, final overall survival was smaller for non‐rescued lines than for rescued lines.

### 3.3 Genomic Patterns of Deleterious Mutations

**FIGURE 6:**
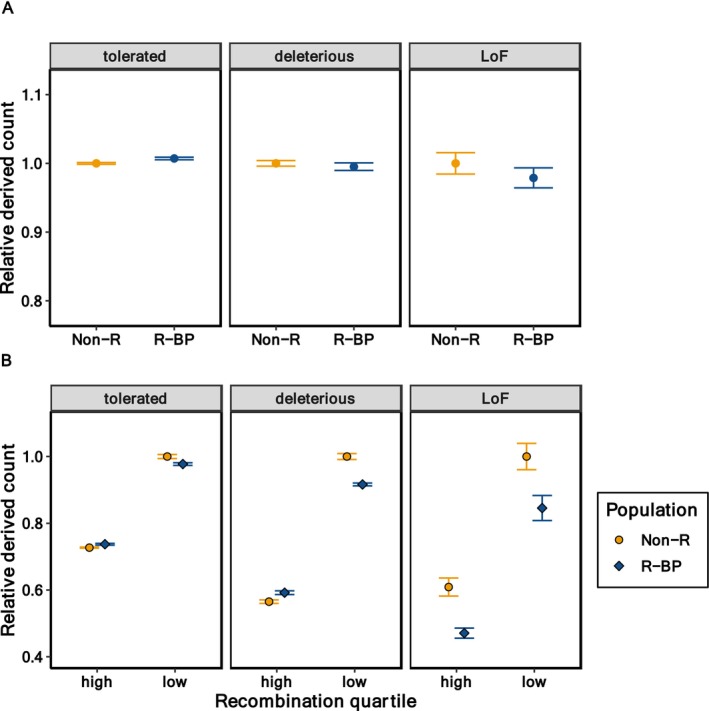
Average derived count of deleterious mutations for different mutation categories for a non‐rescued line (Non‐R) and a rescued‐BP (R‐BP) line. The dots represent the line averages of 12 individuals corrected by the corresponding fourfold synonymous values, and the resulting ratios are relativised by the corresponding non‐rescued line ratios, so that non‐rescued line results always equal one. Error bars represent the standard error of the mean. (A) Whole‐genome analysis. (B) Average derived counts presented separately for high and low recombination genomic regions.

The derived count, corrected by a neutral category, showed a trend toward lower values in the rescued line compared to the non‐rescued line, particularly at deleterious and LoF sites, although these differences were not statistically significant (Figure 6A; detailed results for individual lines are shown in Figure S3). When counts were analysed separately for high and low recombination regions of the genome (Figure 6B), the differences between rescued and non‐rescued lines within each recombination category increased with the predicted severity of deleterious mutations, with LoF mutations showing significantly lower counts in the rescued line across both high and low recombination regions.

Overall, the results suggest that natural selection favoured the beneficial wild‐type alleles introduced during rescue compared to the deleterious derived alleles, and this overcompensated for the introduced inbreeding load that escaped genetic purging.

## 4 Discussion

Although the empirical evidence on the benefits of genetic rescue programs in the short term is extensive (Ralls et al. 2020), some caution should be taken in extreme situations where genetic rescue may lead to an increase in genetic load (Robinson et al. 2019; Wilder et al. 2020; Pérez‐Pereira et al. 2022; Hoy et al. 2023), thus possibly resulting in a higher extinction risk for the rescued population. We performed a laboratory experiment with *D. melanogaster* to assess the ability of a genetic rescue intervention to revert the harmful genetic consequences of reduced size in endangered populations and to improve their survival prospects. The design considers a time horizon that had never been explored so far, because most studies evaluate the effects of genetic rescue only after a few generations and do not look at long‐term consequences (Whiteley et al. 2015; Fitzpatrick et al. 2020). Using a large founded laboratory population (BP), we produced moderately small populations (N50) which were later used to found three sets of small lines (N8) that were either non‐rescued or subjected to genetic rescue using BP or N50 males, and maintained for 33 generations. Our results show an overall success of genetic rescue in consistently increasing population survival through the experiment, particularly in the lines rescued by BP. The factors determining the success of genetic rescue can be multiple and we discuss below the possible role of the more relevant ones.

### 4.1 Hybrid Vigour

Hybrid vigour is considered one of the main advantages of genetic rescue and can be partly responsible for the higher survival of our rescued lines compared to non‐rescued ones. However, we should note that significantly higher productivity in the crosses between rescued‐PB lines compared to the crosses of non‐rescued ones assayed at generation 9 should not be ascribed to pure hybrid vigour produced by rescue, as the first generation of all crosses between independent lines is expected to be similarly hybrid and, therefore, is expected to be similarly vigorous (Wright 1951; Caballero 2020, Chap. 8). Thus, although hybrid vigour may have played some role in the early larger survival of rescued populations, it cannot account for the larger productivity in the crosses of rescued lines. We should also note that our lines had been derived from the N50 populations, which had previously undergone important purging. This should have reduced inbreeding depression in the lines as well as hybrid vigour after rescue.

Regarding the consequences of rescuing using BP or N50 individuals, in the absence of selection, hybrid vigour is expected to halve after one generation of panmixia (Caballero 2020, Chap. 8). In practice, however, natural selection cannot be ignored. First, due to purging in the N50 populations, migrants from this origin can have more wild‐type advantageous copies responsible for hybrid vigour compared to migrants from the base population. In fact, the average productivity for N50 in generation 13 was very close to that of BP, and its inbreeding load was considerably reduced by generation 31. This indicates that these populations had been substantially purged, which should boost the initial increase in fitness in the N50‐rescued lines, conferring some advantage versus rescuing using the BP population. Second, natural selection favours the introduced wild‐type alleles responsible for hybrid vigour. These beneficial alleles are prevalently dominant and are likely to be at low frequency in the rescued lines, which favours efficient selection. Thus, hybrid vigour can fuel future increases in fitness due to natural selection, but probably to a small extent. Despite all these considerations, the survival of rescued‐N50 lines was somewhat lower than that of the rescued‐BP ones. The possible reason for this result is discussed below.

### 4.2 Introduced Inbreeding Load and Genetic Purging

The rescuer individuals can carry many deleterious alleles hidden in heterozygosis that were rare in the donor population. Thus, rescuing introduces inbreeding load, particularly when using large non‐purged donor populations (Hedrick and García‐Dorado 2016). This expectation, however, is not shown by our results, given that at generation 9 and after one generation of inbreeding, the productivity of the rescued‐BP lines was not significantly different from that of the non‐rescued ones (Figure 3). This may be explained by genetic purging removing part of the deleterious alleles introduced by migrants during rescue (García‐Dorado 2012). Thus, although purging is not expected to cause a net increase in fitness compared to the initial average, it can reduce inbreeding depression during the generations following rescue to the point that hybrid vigour and adaptive selection become the main driving forces.

### 4.3 Introduced Adaptive Potential

Finally, we need to consider that our base population was captured from the wild to initiate the experiment, which has been fully performed under laboratory conditions. Therefore, adaptation to the new laboratory conditions may have occurred throughout the experiment.

The average productivity of the BP and N50 populations remained very similar 13 generations after the foundation of the latter (Table 1), which in principle suggests very strong purging in the N50 populations. However, after 18 additional generations, the productivity of the base population increased by a 31% compared to its mean in generation 13, so that the productivity of the N50 population, despite experiencing very little change, became only 72% that of the base population. This is remarkable, as the reduction in productivity in N50 relative to BP due to inbreeding depression is expected to be larger during the first 13 generations than between generations 13 and 31. For example, using the inbreeding load estimated in the base population and assuming an effective size *N*_*e*_ = 25 for the N50 populations (a reasonable assumption following the results from López‐Cortegano et al. 2016), the net decline in productivity (expressed relative to the synchronous base population) between generations 13 and 31 is expected to be only 36% of the net decline produced during the first 13 generations (even less than 36% if purging is considered). However, the decline we obtained from generation 13 to 31 (i.e., 65.58/90.6–67.18/69.18 = 0.247, see Table 1) is 855% the one obtained during the first 13 generations (1–67.18/69.18 = 0.029). A plausible explanation is that the large difference between the productivity of the BP and N50 lines observed at generation 31 could be partly ascribed to more efficient adaptation of the large BP population to the captive laboratory conditions (Orozco‐terWengel et al. 2012), compared to the N50 populations, where rare adaptive alleles are likely to be lost during their foundation. It may be puzzling that this process had no appreciable effect after the first 13 generations and then had such a large effect in the subsequent period of 18 generations. We should note, however, that if adaptive response depends on natural selection favouring alleles that were rare in the large BP population, it should be initially very slow to later accelerate as the frequencies of the adaptive alleles rise enough to produce an increase in additive genetic variance for fitness traits.

The better genetic adaptation to laboratory conditions of the BP population can partially explain the advantage of rescued‐BP over rescued‐N50 lines, due to the introduction of more adaptive variants during rescue. It can also contribute to the higher productivity in the crosses of rescued‐BP lines compared to crosses of non‐rescued lines in generation 9. This possible introgression of alleles responsible for adaptation to captivity can therefore explain the discrepancy between the results of the current experiment and the simulation results from Pérez‐Pereira et al. (2022). In that theoretical work, long‐term rescue of lines kept at small population sizes by a large unpurged population was shown to be unsuccessful, but genetic adaptation was not considered in the simulations. Our present results enlighten the success of genetic rescue in a situation where both the donor and the recipient populations are undergoing an adaptive process due to a common change in the environmental conditions, so that the introduction of adaptive potential from the donor population is likely to be critically relevant to the survival of the endangered one. This situation is of great relevance in conservation biology, particularly when environments are changing rapidly, for example, due to climate change. Finally, our experimental design implies that the investigated lines maintain a constant small size. In real settings, it is expected that the census size would increase over time. If, as hypothesised, selection for adaptation is a main factor for the success of rescuing, the increase in census size would enhance the benefits of rescue.

Additional clues on the relevance of the above causes can be obtained from the molecular analysis of the genomic load. Genomic data currently allow a more detailed assessment of the genetic nature of deleterious variation by enabling a quantification of the number and type of putatively deleterious variants present in a population (Bertorelle et al. 2022). Our results show that the differences between a rescued and a non‐rescued line increased with mutation severity, with LoF counts being significantly lower in the rescued line across both high and low recombination regions. Thus, natural selection seems to have been quite efficient in this rescued line of small size, which is coherent with its larger decline in genetic diversity and a higher inbreeding relative to the non‐rescued one (Table 2). This includes: (i) incorporating beneficial mutations that replace deleterious alleles that were rare in BP and had drifted to large frequencies in the lines; (ii) purging the deleterious load that was unintentionally introduced during rescue; and (iii) incorporating alleles allowing adaptation to captive conditions. This efficiency of natural selection in such small populations enlightens the relevance of large effect variants for the survival of endangered populations. An interesting aspect of our results is that the rescued‐BP line had shorter ROHs than the non‐rescued one, whereas the opposite occurred for long ROHs (> 1.5 Mb) (Figure 4). This may be the consequence of older events of inbreeding that occurred in the BP population (corresponding to short ROH) being introgressed, and possibly selected, in the rescued‐BP line, as well as the breakdown of longer (more recent inbreeding) ROH in the rescued‐BP line due to the introgression of BP males. The molecular analysis carried out has to be taken with caution, though, as only a single non‐rescued line and a single rescued‐BP line were analysed.

The genetic rescue carried out in our study used always males. In practical cases, using female migrants can allow a better control of the amount and variability of inbreeding load introduced, while using males with a mating advantage can boost the short‐term demographic rescue (Zajitschek et al. 2009) but also the spread of the immigrant's inbreeding load, as in the case of the wolf population of Isle Royale ( *C. lupus* ; Hedrick et al. 2014, 2017, 2019; Hoy et al. 2023). A consequence of all migrants having the same sex is that they always mate individuals of the endangered population. The rescue carried out here was restricted to two single generations. Although the extinction rate of the rescued lines was temporally lower than that of the non‐rescued ones, there was a continuous extinction of lines and only a few of them remained after 33 generations: 6 rescued‐BP lines, 3 rescued‐N50 lines and 3 non‐rescued lines. The theoretical results obtained by Pérez‐Pereira et al. (2022) support that a continuous stable connection between populations is more effective than punctual or sporadic recurrent rescue. In fact, continuous migration has been recently recommended for the reintroduced Eurasian lynx populations ( *Lynx lynx* ), given the levels of inbreeding and genetic diversity observed (Mueller et al. 2022). Moreover, Armstrong et al. (2021) pointed to facilitating gene flow between populations as a possible solution to reduce the impact of inbreeding after reintroduction, although they noted that its consequences would be complex and not easily predicted. Finally, Smeds and Ellegren (2023) have also indicated that continuous reconnection is safer than sporadic ones. The highly inbred Scandinavian wolf ( *C. lupus* ), founded by only three wolves in the 1980s was rescued by four immigrants in 2008–2013. These gave a temporary genetic rescue effect but in the absence of more migration events, inbreeding led again to the exposure of deleterious mutations (Smeds and Ellegren 2023).

The number of reintroduced individuals and its origin is also relevant. Simulation results by Al Hikmani et al. (2024) using the critically endangered Arabian leopard as a model species showed that the optimal regular reintroduction from a captive population to an endangered wild one would be of two individuals every generation (every 5 years in the model). This would improve the long‐term viability of the wild population by reducing its genetic load. Reintroducing more than two individuals, however, would imply a genetic erosion of the wild population, with negative consequences, although it must be stressed that in this case the genetic rescue was assumed to be made from a captive population maintained with only 64 individuals, rather than with a large wild population.

Recent studies have yielded contrasting results regarding how long the positive effects of genetic rescue are maintained, particularly after a single migration event. Several experimental results support the cautions raised by Pérez‐Pereira et al. (2022) regarding the risk of introducing load during genetic rescue into too small populations. For example, Ochoa et al. (2022) recently warned about the potential cost of genetic rescue in the Florida panther ( *Puma concolor* ), because of the introduction of deleterious alleles after migration that could be expressed due to increased homozygosity by inbreeding if the population size remains small or suffers additional bottlenecks. Malmberg et al. (2019) also warned about the transmission of viruses during translocations with this species. Dussex et al. (2021) and Foster et al. (2021) also warned about the possible risk of introducing detrimental alleles into the island population of the critically endangered owl parrot kākāpō ( *Strigops habroptilus* ), based on the larger load of deleterious alleles detected in continental individuals and the currently small effective population size, and given the current recommendation to promote matings between continental and insular descendants. Hasselgren et al. (2018) showed results for a natural population of Scandinavian arctic foxes ( *Vulpes lagopus* ), which was (re)founded by six individuals and remained isolated at an extremely low population size for 9 years. In 2010, the population was subject to the natural migration of three males (released from a captive breeding programme), which initially caused a genetic rescue effect. However, 20 years of pedigree data, combined with microsatellite information, found no evidence of increased fitness in generations F2 and F3, a (fluctuating) trend of increased inbreeding after migration, a 22% reduction in population size about 9 years after migration, and an increase in migrant ancestry up to 27% (Lotsander et al. 2021). Molecular data from genome sequencing showed an increase in inbreeding in the first 3 years after migration to values close to those found before migration, and heterozygosity was significantly reduced in the F2 and F3 generations (Hasselgren et al. 2021).

Our study was carried out with a model species, *D. melanogaster* , which allows a long‐term screening of genetic rescue, difficult to achieve in natural populations. Jørgensen et al. (2022) also conducted a study with inbred lines of *D. melanogaster,* covering up to four generations after the rescue program. Their results add to the previous evidence on the positive effect of rescue programs on fitness, an effect that remained until the fourth generation. They founded multiple inbred lines from a large laboratory population, which were subjected to three consecutive generations of full‐sib mating (i.e., fast inbreeding) prior to the rescue treatment. The inbred lines showing the lowest median egg‐to‐adult viability were used as recipient populations, and those with the highest median were used as donor populations, which implies that the donor populations were chosen so that they may have undergone some successful purging, at least for severely deleterious alleles, while recipient populations seemed to have undergone little purging, as is likely to occur under such fast inbreeding. One aspect to note is that, even when sharing the same origin and inbreeding history, there is a great stochasticity linked to the inbreeding load inherited by each line from the large population, resulting in considerable variation in the magnitude of inbreeding depression (as observed by Jørgensen et al. 2022). The fraction of (partially) recessive deleterious alleles fixed by drift in each line is also expected to differ. However, genetic rescue is expected to be done using large outbred or slowly inbred donor populations, if available, rather than inbred lines. Therefore, in addition to the hybrid vigour induced by the masking of fixed deleterious mutations, the positive result observed for genetic rescue in this study could have been favoured by the possible purging of some alleles of large deleterious effect in the donor populations during the period of fast inbreeding.

We have illustrated a situation where genetic rescue was successful at reducing extinction risk in populations that remained small for a long period after the rescue in a situation where both donor and recipient populations are likely to be undergoing a common adaptive process. Our results suggest that this is mainly due to the power of natural selection to modulate the variability introduced during rescue, even in very small populations. Although this includes the incorporation of wild‐type beneficial alleles that had been lost by drift in the lines with small population sizes and some purging of inbreeding load introduced during genetic rescue, it seems that the incorporation of alleles allowing adaptation to the new laboratory environment may have played a major role in determining the success of genetic rescue. This enlightens the usefulness of genetic rescue as a mechanism to increase adaptive potential, which can be particularly high when both the donor and the recipient populations are undergoing adaptation to similar environmental challenges. In such circumstances, we see that rescue is more efficient when using larger donor populations, as expected from their larger adaptive potential and their expected better ability to adapt. A parallel practical example can be the rescue of small populations adapting to global challenges, such as climate change. The success of rescue programmes, however, will obviously depend on the particular scenarios involved, and universal recommendations about its application cannot be given.

## Author Contributions

Noelia Pérez‐Pereira, Aurora García‐Dorado, and Armando Caballero designed the study. Noelia Pérez‐Pereira carried out the fly experiments. Daniel Kleinman‐Ruiz and Humberto Quesada processed the genomic data and carried out the genomic analyses. All authors contributed to the analyses of results and participated in interpreting and discussing them. All authors contributed to the writing of the manuscript and approved the final version.

## Conflicts of Interest

The authors declare no conflicts of interest.

## Supporting information


Data S1.

## Data Availability

Scripts are available at Github address https://github.com/noeliaperezp/Genetic‐rescue‐Drosophila‐melanogaster. FASTQ files have been deposited in the NCBI Sequence Read Archive (SRA) database under accession numbers SAMN44484872 –SAMN44484895 (BioProject accession: PRJNA1178870).
