# Supplementary material for: A Test of the Long‐Term Efficiency of Genetic Rescue With Drosophila melanogaster
Source: Mol Ecol. 2025 Feb 18;34(23):e17690. doi: 10.1111/mec.17690 (PMC12684308; doi:10.1111/mec.17690)
Supplement: Supplementary file 1 — Data S1. [file MEC-34-e17690-s001.docx]

**Supplemental Information for:**

**A test of the long term efficiency of genetic rescue with *Drosophila melanogaster***

Noelia Pérez-Pereira, Daniel Kleinman-Ruiz, Aurora García-Dorado, Humberto Quesada and Armando Caballero

**Table of Contents:**

| **Table S1** | Page 2 |
| --- | --- |
| **Table S2** | Page 3 |
| **Figure S1** | Page 4 |
| **Figure S2** | Page 5 |
| **Figure S3** | Page 6 |

**Table S1.** Average number of ROH per individual and their average length for the 12 individuals of the non-rescued line and the 12 individuals of the rescued-BP line, after changing some of the parameters to detect ROH with the software PLINK.

| **Density** | **Gap** | **Win-size** | **Win-Thres** |  | **Num. ROH** | **Length ROH** |
| --- | --- | --- | --- | --- | --- | --- |
| **Non-Rescued** | |  |  |  |  |  |
| 50 | 50 | 30 | 0.05 |  | 105.67 | 421.62 |
| **200** | 50 | 30 | 0.05 |  | 105.67 | 421.62 |
| 50 | **200** | 30 | 0.05 |  | 111.58 | 428.70 |
| 50 | 50 | 30 | **0.01** |  | 83.67 | 546.07 |
| 50 | 50 | **60** | 0.05 |  | 107.33 | 407.57 |
| **Rescued-BP** | |  |  |  |  |  |
| 50 | 50 | 30 | 0.05 |  | 121.08 | 408.52 |
| **200** | 50 | 30 | 0.05 |  | 121.08 | 408.52 |
| 50 | **200** | 30 | 0.05 |  | 124.83 | 429.49 |
| 50 | 50 | 30 | **0.01** |  | 99.08 | 514.33 |
| 50 | 50 | **60** | 0.05 |  | 121.17 | 397.53 |

Density: minimum number of kilobases per SNP. Gap: homozygous gap in kb. Win-size: scanning window in number of SNPs. Win-Thres: threshold window.

**Table S2.** Rates of extinction of the lines in percentage, averaged for each third of the experiment.

| Generations | Non-rescued | Rescued-PB | Rescued-N50 |
| --- | --- | --- | --- |
| 1 - 11 | 0.1162 | 0.0700 | 0.0971 |
| 12 - 22 | 0.1075 | 0.1131 | 0.0899 |
| 23 - 33 | 0.0333 | 0.0492 | 0.0587 |

**Figure S1.** Design of experiments to estimate pupae productivity (*P*) in outbred and inbred individuals of the small lines with *N* = 8 individuals. At generation 9 of the lines, non-rescued and rescued-BP lines were evaluated for pupae productivity following two schemes, one crossing individuals from different lines, and another carrying out full-sib mating for two consecutive generations.

**
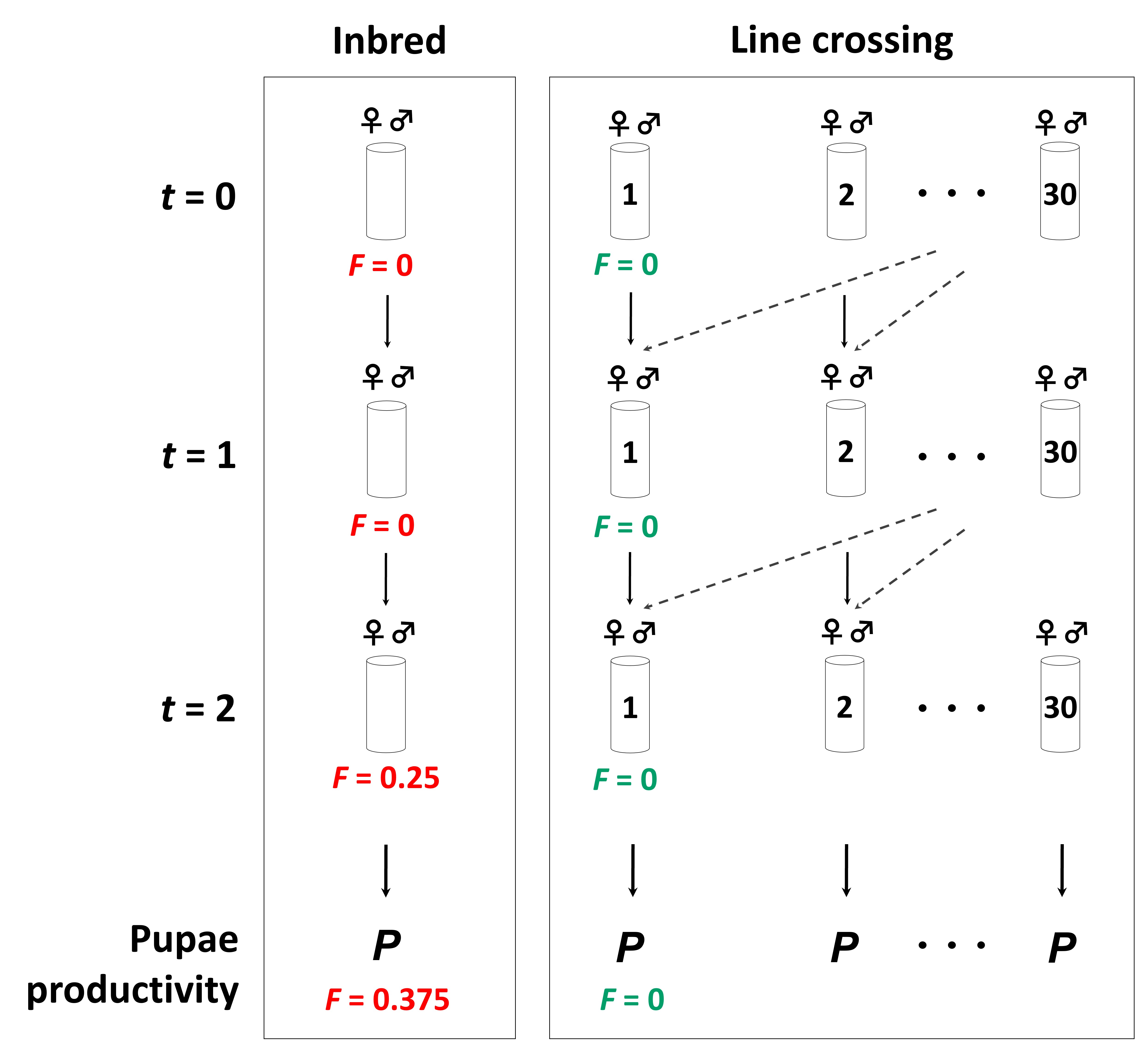
**

**Figure S2.** Coefficient of inbreeding of the 12 individuals in the non-rescued (Non-R) line and the 12 individuals in the rescued-BP (R-BP) line, obtained from Runs-of-Homozygosity (ROH) with lengths larger than 100 kb.

**
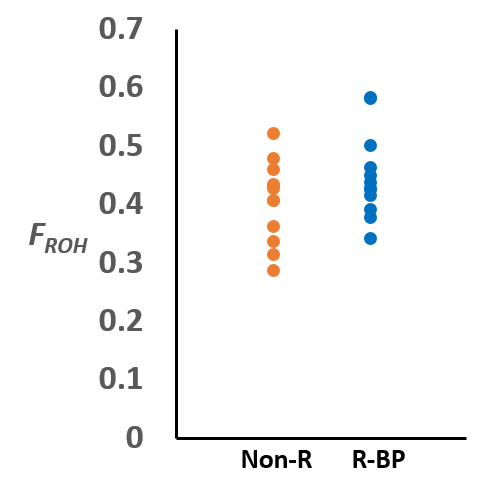
**

**Figure S3.** Relative derived count of deleterious mutations for different mutation categories for a non-rescued line (Non-R) and a rescued-BP (R-BP) line. ﻿The dots represent the values for each of the 12 individuals corrected by the corresponding fourfold synonymous values, and the resulting ratios are relativized by the corresponding non-rescued line ratios, so that non-rescued lines results always equal one.

**
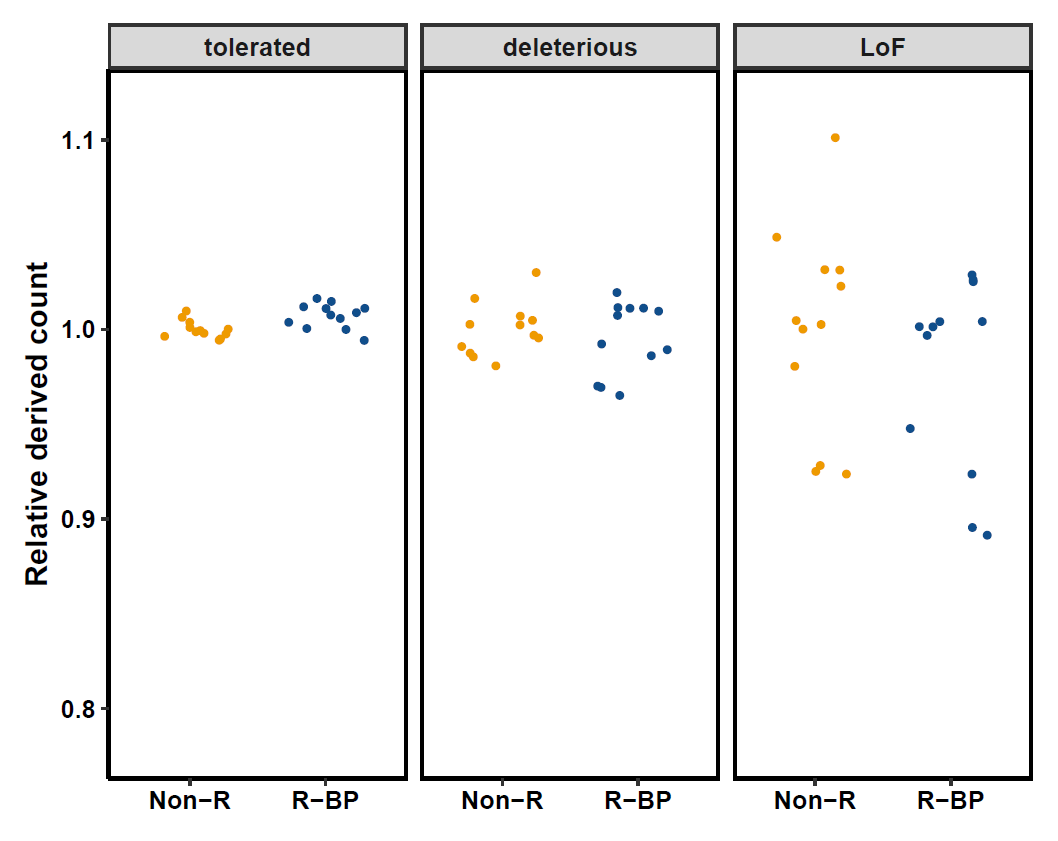
**
